# Acute and Chronic Changes in Gene Expression After CMV DNAemia in Kidney Transplant Recipients

**DOI:** 10.3389/fimmu.2021.750659

**Published:** 2021-11-15

**Authors:** Richard Ahn, Joanna Schaenman, Zachary Qian, Harry Pickering, Victoria Groysberg, Maura Rossetti, Megan Llamas, Alexander Hoffmann, David Gjertson, Mario Deng, Suphamai Bunnapradist, Elaine F. Reed, CMV Systems Immunobiology Group, Richard Ahn

**Affiliations:** ^1^ Department of Microbiology, Immunology, and Molecular Genetics, University of California Los Angeles, Los Angeles, CA, United States; ^2^ Institute for Quantitative and Computational Biosciences, University of California Los Angeles, Los Angeles, CA, United States; ^3^ Department of Medicine, University of California Los Angeles, Los Angeles, CA, United States; ^4^ Department of Pathology and Laboratory Medicine, University of California Los Angeles, Los Angeles, CA, United States; ^5^ Department of Biostatistics, University of California Los Angeles, Los Angeles, CA, United States

**Keywords:** kidney transplant, RNA-seq, CMV DNAemia, transplant immunology, transcriptomics

## Abstract

Cytomegalovirus (CMV) viremia continues to cause significant morbidity and mortality in kidney transplant patients with clinical complications including organ rejection and death. Whole blood gene expression dynamics in CMV viremic patients from onset of DNAemia through convalescence has not been well studied to date in humans. To evaluate how CMV infection impacts whole blood leukocyte gene expression over time, we evaluated a matched cohort of 62 kidney transplant recipients with and without CMV DNAemia using blood samples collected at multiple time points during the 12-month period after transplant. While transcriptomic differences were minimal at baseline between DNAemic and non-DNAemic patients, hundreds of genes were differentially expressed at the long-term timepoint, including genes enriching for pathways important for macrophages, interferon, and IL-8 signaling. Amongst patients with CMV DNAemia, the greatest amount of transcriptomic change occurred between baseline and 1-week post-DNAemia, with increase in pathways for interferon signaling and cytotoxic T cell function. Time-course gene set analysis of these differentially expressed genes revealed that most of the enriched pathways had a significant time-trend. While many pathways that were significantly down- or upregulated at 1 week returned to baseline-like levels, we noted that several pathways important in adaptive and innate cell function remained upregulated at the long-term timepoint after resolution of CMV DNAemia. Differential expression analysis and time-course gene set analysis revealed the dynamics of genes and pathways involved in the immune response to CMV DNAemia in kidney transplant patients. Understanding transcriptional changes caused by CMV DNAemia may identify the mechanism behind patient vulnerability to CMV reactivation and increased risk of rejection in transplant recipients and suggest protective strategies to counter the negative immunologic impact of CMV. These findings provide a framework to identify immune correlates for risk assessment and guiding need for extending antiviral prophylaxis.

## 1 Introduction

Despite years of improvements in screening, prophylaxis, and treatment options, CMV continues to cause significant negative impact in solid organ transplant recipients, *via* both direct and indirect effects (1). Direct effects include disseminated infection and end-organ disease including pneumonitis, esophagitis, and colitis. Indirect effects, in contrast, cause more impactful consequences of CMV infection, including vulnerability to secondary infections and development of alloimmunity, leading to allograft dysfunction. Heterologous immunity has been postulated as the mechanism behind this negative effect on the allograft, although generalized increase in inflammation is another possible cause (2, 3). Evaluation of the transcriptional changes secondary to CMV primary infection or reactivation of CMV can provide insight into the mechanisms behind allograft dysfunction secondary to CMV.

Previous studies on host response to CMV are primarily derived from the MCMV mouse model. These have demonstrated upregulation of *Cxc3r1*, *Cd69*, and *Cd103*, associated with inflation of T cell memory, tissue association, and exhaustion, as well as transcription factors associated with inflammation (4, 5). This shift towards CMV-specific memory T cells is postulated to underlie the progression to increased immune senescence (6). Another key aspect of CMV infection is the establishment of latent infection and virus-mediated downregulation of host genes, blocking ability to clear the virus (7). However, less is known in the context of human CMV infection.

Solid organ transplantation presents a unique clinical model to investigate CMV infection in humans, where the serostatus of both donor and recipient is known prior to transplantation, and CMV PCR is followed for surveillance purposes after transplantation, allowing for identification of the initiation of primary CMV infection or reactivation. This provides an opportunity to examine changes in host gene expression before and after the start of detectable CMV DNAemia, providing insights into the impact of CMV infection on the transplant recipient’s innate and adaptive antiviral immune response and potential influence on graft outcome. We therefore characterized the whole blood transcriptome from a cohort of kidney transplant recipients over several time points during and after detectable CMV infection to analyze changes in gene expression over time, as well as differences between patients with or without CMV DNAemia.

## 2 Methods

### 2.1 Patient Recruitment and Clinical Care

Kidney transplant recipients were enrolled in a UCLA IRB-approved study, and blood was collected at regular intervals after transplantation followed by PBMC isolation and storage, as previously described (8). Patients received induction with either anti-thymocyte globulin (ATG) or basiliximab depending on pre-transplant levels of sensitization and donor kidney quality followed by protocol-based immunosuppression with tacrolimus, mycophenolate mofetil, and prednisone, as previously described (8). Patients intolerant of tacrolimus were switched to cyclosporine. CMV prevention was performed according to local protocols as previously described, summarized as follows: 6 months of valganciclovir for high-risk donor positive (D+) and recipient negative (R−) patients and 3 months of valganciclovir for intermediate-risk recipient positive (R+) patients who received ATG induction. R+ patients who received basiliximab or low-risk (D−/R−) patients received acyclovir prophylaxis to prevent HSV and VZV infection. All patients regardless of prophylaxis regimen underwent regular CMV PCR screening to detect CMV DNA in peripheral blood for the first year after transplantation.

### 2.2 Patient Selection

Patients with a history of positive testing by CMV DNA by PCR were identified by review of microbiology records. We reviewed our repository database to determine whether PBMCs were available that corresponded to the following timepoints: (1) Baseline (prior to DNAemia start), (2) 1 week post-DNAemia start (week 1), (3) 1 month post-DNAemia (month 1), and (4) ~1 year after transplantation (long-term). Research blood was collected from stable outpatients at the time of kidney transplant clinic visit. Four of the 31 patients that developed CMV DNAemia after transplantation were donor IgG seropositive, recipient seronegative; the others were recipient IgG seropositive. For patients with multiple episodes of CMV DNAemia over 137 IU/ml, the first episode closest to transplantation was studied.

Patients with history of CMV DNAemia were matched on a 1:1 basis to a cohort of kidney transplant recipients without history of CMV DNAemia based on deceased *versus* living donor status, patient age, sex, race/ethnicity, and induction type with kidney transplant recipients who were either D+/R− or R+. Samples were selected for each control patient that corresponded in terms of time post-transplant with the baseline and long-term CMV DNAemia patient samples. Of these patients negative for CMV DNAemia, 24 were CMV seropositive and 7 were seronegative with CMV-positive donors. Antiviral prophylaxis was administered by protocol, so that high-risk patients (D+/R−) received 6 months of Valcyte, and intermediate-risk patients (R+) received 3 months of Valcyte if they received antithymocyte globulin induction, otherwise acyclovir for 3 months, as previously described (9). Patients with detectable levels of CMV DNAemia >137 received antiviral therapy per protocol for a minimum of 2 weeks, and until CMV PCR was <137, per American Society of Transplantation guidelines (1). We focused exclusively on CMV infection as this was our central scientific question, and due to the fact that other post-transplant infections are either much rarer compared with CMV, the most common viral infection after kidney transplantation (10), or site specific and limited to a single organ such as the case for urinary tract infection, and therefore predicted to have a much less significant impact on peripheral blood transcriptional analysis compared with CMV.

### 2.3 RNA Sequencing

#### 2.3.1 Sample Preparation and Sequencing

RNA was isolated from 1 ml RBC-lysed whole blood samples stored in RNAlater (500 μl). Globin RNA was removed using the GLOBINCLEAR kit. Libraries were prepared for samples that passed quality control using the KAPA stranded mRNA kit. Single-read sequencing with read length of 50 bp was performed using the Illumina HiSeq3000 platform.

#### 2.3.2 Read Quality Control and Alignment

After sequencing, quality control of reads was checked using FastQC 0.11.8 (11). Reads were aligned to the GRCh38 human reference genome using STAR 2.4.2 (12). We used samtools (13) and picard (https://github.com/broadinstitute/picard) to index the resulting BAM files and remove any PCR duplicates. Ten samples were removed from further downstream analysis because of low library size and/or a high proportion of reads that did not uniquely map to the reference. We used featureCounts (14) to produce the final count table.

### 2.4 Statistical Analysis

#### 2.4.1 Univariate Statistical Analysis

To test for association between demographic and clinical data and CMV DNAemia status, Fisher’s exact test was implemented for categorical variables, while Welch’s two-sample t-test was implemented for continuous variables.

#### 2.4.2 Differential Expression Analysis and Pathway Analysis

We used the edgeR 3.9 package (15) in R (16) to convert the raw counts to counts-per-million (CPM), to perform trimmed mean of M-values normalization, and to test for differential expression by fitting a negative binomial generalized linear model, estimate dispersion estimates, and perform a likelihood ratio test. We tested for differential expression between (1) baseline and week 1, (2) baseline and month 1, (3) baseline and long-term, (4) CMV DNAemia *vs* no CMV DNAemia at baseline, and (5) CMV DNAemia *vs* no CMV DNAemia at long-term. We used Ingenuity Pathway Analysis (IPA) to perform canonical pathway enrichment analysis of the differentially expressed genes (DEGs).

#### 2.4.3 Time-Course Gene Set Analysis

We implemented the TcGSA R package (17) to perform time-course gene set analysis to longitudinally test the stability of gene set expression. In summary, for each gene set, TcGSA implements a linear mixed model of gene expression that includes a fixed-effect for the expression of each gene in a gene set, a random-effect of the expression of each gene in a gene set from each patient, as well as a time function to model the time trend for each gene. This time function can be divided into a fixed effect that represents the average trend of the gene set and a random effect that accounts for the heterogeneity that may be present between each gene and the gene set and may take a linear polynomial, cubic polynomial, or natural cubic spline form. For this study, a cubic polynomial form was chosen for the time function as it provided a better fit to the data than a linear polynomial form but an equivalent fit to the natural cubic spline form but with fewer degrees of freedom. After modeling the gene expression in each gene set, a likelihood ratio test was implemented to test whether a gene set had a significant time trend, where the null hypothesis was that the expression genes in a gene set were stable over time.

## 3 Results

### 3.1 Patient Demographics

A summary of patient demographics is provided in **Table 1**. As expected, due the propensity-matching design for patient selection, age, sex, race, and induction type were comparable between patients with DNAemia (≥137 IU/ml of CMV detected in a PCR test; abbreviated PCR+) and non-DNAemic patients (PCR−) (**Table 1**). A similar number of patients were high risk (D+/R−) as intermediate risk (R+) in both groups, and median GFR at 3 months and rates of rejection were also similar between groups. Patients experiencing DNAemia were PCR-positive at a median of 80 days after transplantation (range: 10 to 561 days). Median peak viral load was 757 IU/ml (range 146 to 13,900 IU/ml). Two patients with DNAemia were diagnosed with CMV syndrome or CMV end-organ disease based on standard definitions (18).

**Table 1:** Demographic and clinical characteristics of kidney transplant recipients with and without detectable CMV viremia.

	CMV DNAemia (n = 31)	No CMV DNAemia (n = 31)	p-value
**Race [n (%)]**			0.41
White	7 (22.6%)	12 (38.7%)	
Black/African-American	7 (22.6%)	3 (9.7%)	
LatinX/Hispanic	9 (29.0%)	9 (29.0%)	
Other	8 (25.8%)	7 (22.6%)	
**Gender [n (%)]**			0.79
Female	10 (32.3%)	12 (38.7%)	
Male	21 (67.7%)	19 (61.3%)	
Age^*^ [median (IQR)]	55 (14)	53 (16.5)	0.99
**Kidney disease [n (%)]**			0.27
Diabetes mellitus	8 (25.8%)	11 (35.5%)	
Glomerulonephritis	10 (32.3%)	5 (16.1%)	
Hypertension	5 (16.1%)	3 (9.7%)	
Polycystic Kidney Disease	1 (3.2%)	5 (16.1%)	
Other	7 (22.6%)	7 (22.6%)	
**Donor type [n (%)]**			0.80
Deceased	17 (54.8%)	15 (48.4%)	
Living	14 (45.2%)	16 (51.6%)	
**Diabetes [n (%)]**			0.43
No	22 (71.0%)	18 (58.1%)	
Yes	9 (29.0%)	13 (41.9%)	
**Induction with ATG [n (%)]**			>0.99
No	22 (71.0%)	22 (71.0%)	
Yes	9 (29.0%)	9 (29.0%)	
GFR at 6 months^*^ [median (IQR)]	50.1	52.2	0.6
GFR at 1 year^*^ [median (IQR)]	50.7	54.3	0.41
**Rejection at 1 year**			0.26
No	25 (80.6%)	29 (93.5%)	
Yes	6 (19.4%)	2 (6.5%)	
**Graft status at 1 year**			>0.99
Functioning	31 (100.0%)	31 (100.0%)	
**Duration of DNAemia in days [median (IQR)]**	21 (15)	-	-
**Time in days from transplant to: [median (IQR)]**			
Baseline	55 (20)	90 (74)	–
Week 1	92 (115)	-	
Month 1	176 (189)	–	
Long-term	339 (93)	362 (62)	-

*p-value from Welch’s two-sample t-test.

### 3.2 Transcriptomics of DNAemic Patients Over Time: Individual Gene Analysis

To characterize the transcriptional landscape of CMV DNAemia patients across a 1-year timeframe post-transplant, we performed RNA-seq on whole blood from 31 patients with CMV DNAemia, across the four timepoints (n=131) described above. Both differential expression analysis and pathway analysis were performed for each time point.

**Figure 1:**
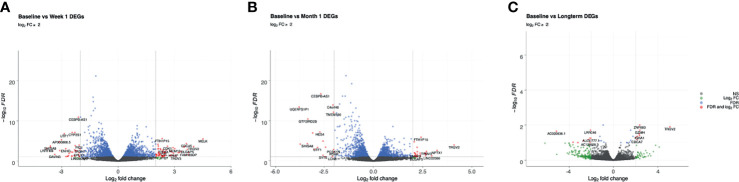
Volcano plots of genes that are differentially expressed between **(A)** baseline and week 1; **(B)** baseline and month 1; **(C)** baseline and long-term in patients that developed DNAemia. Gray points represent genes that are not differentially expressed, green points represent genes that have a log_2_FC ≥ 2 but not differentially expressed, blue points represent genes that are differentially expressed (FDR ≤ 0.1) but have a log_2_FC < 2, and red points represent genes that are both differentially expressed (FDR ≤ 0.1) and have a log_2_FC ≥ 2.

Review of differential expression of all genes with FDR<0.1 demonstrated striking changes after detection of CMV DNAemia. Analysis of the difference between baseline and week 1 post-DNAemia revealed the most prominent transcriptional changes with respect to the number of DEGs, with 2,456 DEGs total and 538 of those DEGs having an absolute log fold-change (|logFC|) ≥ 1 at FDR ≤ 0.1 (**Figure 1A** **;** **Table 2A**; see **Supplementary Data 1** for full listing of all DEGs). A total of 1,299 DEGs were upregulated and 1,157 downregulated at week 1 after CMV DNAemia compared with baseline (**Table 2A**). Several of these DEGs encompass genes that are involved in immune signaling, cytotoxicity, and T cell activation, such as *TRGV2* (T Cell Receptor Gamma Variable 2) and *TRDV2* (T Cell Receptor Delta Variable 2), which are upregulated, and *IL1A* (Interleukin 1 Alpha), which is downregulated (top 20 up-/downregulated genes are shown in **Table 2B**). Besides these notably higher transcriptional changes, we detected subtle variations in several immunologically relevant genes at week 1 post-viremia, including transcripts encoding for transcription factors *HOPX* (Homeodomain-only protein) and *BATF2* (Basic Leucine Zipper ATF-Like Transcription Factor 2) were slightly upregulated 1 week after CMV DNAemia (**Supplementary Data 1**). In support of potential ongoing TCR engagement, we found an increase of *CD69* and a simultaneous expression of TCR dependent co-inhibitory molecules of *LAG3* (Lymphocyte Activating 3) and *CTLA4* (Cytotoxic T-lymphocyte Associated Protein 4) at 1 week post-DNAemia. In addition, we found that transcripts encoding *DUSP4* (Dual Specificity Phosphatase 4)*, KLRG1* (Killer Cell Lectin Like Receptor G1), *CD70* were also enriched, which are linked to inhibitory function and to negative regulation of T cell activation and proliferation.

**Table 2A:** Differential expression results for patients with DNAemia.

Comparison (A *vs* B)	Total DEGs*	Upregulated in B	Downregulated in B	|log_2_FC| ≥ 1
**Baseline *vs* week 1**	2,456	1,299	1,157	538
**Baseline *vs* month 1**	1,972	1,030	255	280
**Baseline *vs* long-term**	29	15	14	26

*FDR ≤ 0.1.

**Table 2B:** Top 20 up-/downregulated genes from each timepoint comparison for patients with DNAemia.

Baseline *vs* week 1			Baseline *vs* month 1			Baseline *vs* long-term		
Gene	log_2_FC	FDR	Gene	log_2_FC	FDR	Gene	log_2_FC	FDR
	*Upregulated*			*Upregulated*			*Upregulated*	
*MELK*	4.51	3.03E-06	*TRGV2*	4.10	1.01E-04	*TRGV2*	5.06	1.24E-02
*TRGV2*	4.02	2.49E-04	*NPTX1*	3.23	2.23E-03	*GZMH*	2.38	1.93E-02
*FAM183DP*	3.75	7.77E-03	*LINC02086*	2.96	5.53E-02	*ZNF683*	2.37	9.82E-03
*KIF4A*	3.74	2.51E-02	*TRDV3*	2.69	4.03E-03	*IGHA1*	2.35	3.94E-02
*DLGAP5*	3.68	1.24E-03	*HAMP*	2.68	1.76E-02	*PDGFD*	2.32	3.16E-02
*CDC45*	3.63	4.49E-05	*IGFL2*	2.67	1.63E-02	*FCRL6*	2.14	4.36E-02
*IQSEC3*	3.61	2.61E-02	*CCL4*	2.65	5.17E-02	*CDCA7*	2.12	6.64E-02
*KIF2C*	3.55	2.22E-04	*NUF2*	2.63	1.07E-02	*FGFBP2*	2.01	5.06E-02
*NPTX1*	3.49	3.29E-04	*MATN2*	2.62	1.09E-02	*ADGRG1*	1.76	4.36E-02
*CCNB2*	3.35	4.26E-03	*FTH1P15*	2.42	1.21E-06	*HOPX*	1.56	7.36E-02
*CDKN3*	3.26	6.68E-03	*CD8B2*	2.38	3.13E-02	*C1orf21*	1.55	1.93E-02
*NUF2*	3.19	7.79E-03	*RASD2*	2.37	8.09E-02	*CCL5*	1.45	9.45E-02
*TRDV2*	3.08	6.50E-02	*ORM2*	2.37	1.10E-02	*GZMA*	1.23	1.00E-01
*MCM10*	3.01	7.95E-04	*ZNF681*	2.24	7.61E-02	*MATK*	1.20	9.88E-02
*HAMP*	2.94	1.11E-02	*IFNG*	2.23	4.77E-03	*DCTD*	0.67	9.88E-02
*KIF15*	2.93	4.84E-02	*UBXN10*	2.23	2.15E-02	–	–	–
*CENPM*	2.92	4.43E-04	*AC115223.1*	2.22	3.77E-04	-	-	-
*AC100835.2*	2.86	5.36E-03	*TRBV11-2*	2.20	6.39E-02	–	–	–
*GPR19*	2.82	2.85E-02	*DLGAP5*	2.18	9.67E-02	-	-	-
*SKA3*	2.79	4.10E-02	*HBG1*	2.16	7.25E-02	–	–	–
	*Downregulated*			*Downregulated*			*Downregulated*	
*AP003354.2*	−2.34	5.19E-02	*UNC93B2*	−1.94	1.11E-05	–	–	–
*ALOX15B*	−2.42	5.84E-03	*CEBPD*	−1.96	9.50E-02	-	-	-
*SRXN1*	−2.44	7.96E-08	*PDXP*	−2.01	3.55E-04	–	–	–
*IL1A*	−2.46	6.72E-02	*PCSK1N*	−2.03	7.94E-02	-	-	-
*AL391807.1*	−2.46	5.24E-02	*TMEM160*	−2.03	1.19E-14	–	–	–
*FMN1*	−2.50	5.48E-03	*C4orf48*	−2.06	7.64E-13	-	-	-
*HELLPAR*	−2.61	1.25E-02	*LCN8*	−2.09	1.36E-03	*FCHO2*	−0.83	9.45E-02
*NEBL*	−2.63	4.28E-02	*AC103810.3*	−2.44	5.63E-03	*FAM126B*	−0.90	9.82E-03
*PKD1P5*	−2.67	6.77E-02	*XIST*	−2.44	2.29E-08	*ADCY4*	−1.06	7.78E-02
*AC093274.1*	−2.70	1.04E-02	*AC103591.3*	−2.56	8.88E-02	*GPR84*	−1.19	4.36E-02
*NRCAM*	−2.77	7.83E-02	*SYT5*	−2.57	3.61E-02	*CLDN9*	−1.20	7.78E-02
*ENHO*	−2.78	8.10E-04	*CEBPB-AS1*	−2.68	2.08E-17	*AL031777.1*	−2.01	5.54E-02
*UTF1*	−2.86	1.60E-07	*HES4*	−2.73	6.18E-08	*LRRC46*	−2.09	1.93E-02
*AP000866.5*	−3.01	5.20E-06	*UTF1*	−2.85	4.05E-04	*AC138028.3*	−2.13	9.45E-02
*AC103996.2*	−3.11	2.31E-03	*AL365226.2*	−3.00	1.22E-03	*CTBP1-AS*	−2.13	5.50E-02
*GTF2IRD2B*	−3.13	1.62E-05	*AC145285.6*	−3.09	1.02E-07	*AC068580.3*	−2.28	6.64E-02
*CAVIN3*	−3.37	2.27E-02	*GTF2IRD2B*	−3.34	3.66E-11	*AL590385.2*	−2.74	7.78E-02
*SHISA8*	−3.60	1.76E-04	*SHISA8*	−3.36	4.85E-05	*CNN1*	−3.22	9.88E-02
*SYT5*	−3.61	3.55E-04	*AP000866.5*	−3.72	6.16E-05	*SERPINB2*	−3.64	9.45E-02
*LRRTM4*	−3.79	7.01E-04	*UQCRFS1P1*	−3.78	3.84E-14	*AC020636.1*	−5.13	2.21E-02

While many of these genes a showed transient change in expression, several molecules showed a stable pattern of expression emerging week 1 and remained detectably higher at the month 1 and long-term timepoints. One such molecule is *FCRL6* (Fc Receptor Like 6) (**Table 2B** *)*, a distinct marker of cytotoxic effector lymphocytes. The other gene is *GPR56*, a marker associated with CD56null/CD16+ NK cells. Interestingly, we also noted decrease in *IRF8*, which orchestrates adaptive NK-cell responsiveness and antiviral immunity.

Analysis of differentially expressed genes at 1 month after CMV DNAemia compared with baseline revealed ongoing changes in pathways important for immunologic function, with a total of 1,972 significantly enriched DEGs (**Figure 1B** **;** **Table 2A**). These included upregulation of genes important to adaptive and innate immune function including *TRGV2*, *TRDV3* (T Cell Receptor Delta Variable 3), *IGFL2* (IGF Like Family Member 2), *CCL4* (C-C Motif Chemokine Ligand 4), and *IFNG* (Interferon-γ) (**Table 2B**).

At the long-term timepoint, there were fewer changes in canonical pathways compared with the week 1 to baseline change; nonetheless, 29 statistically significant differences in DEGs were detected (**Figure 1C** **;** **Table 2A**). Genes from within these DEGs included upregulation in *TRGV2*, *GZMH*, *GZMA*, *FCRL6* (Fc Receptor Like 6), *and CCL5* (Chemokine Ligand 5) (**Table 2B**).

### 3.3 Transcriptomics of DNAemic Patients Over Time: Pathway Analysis

**Figure 2:**
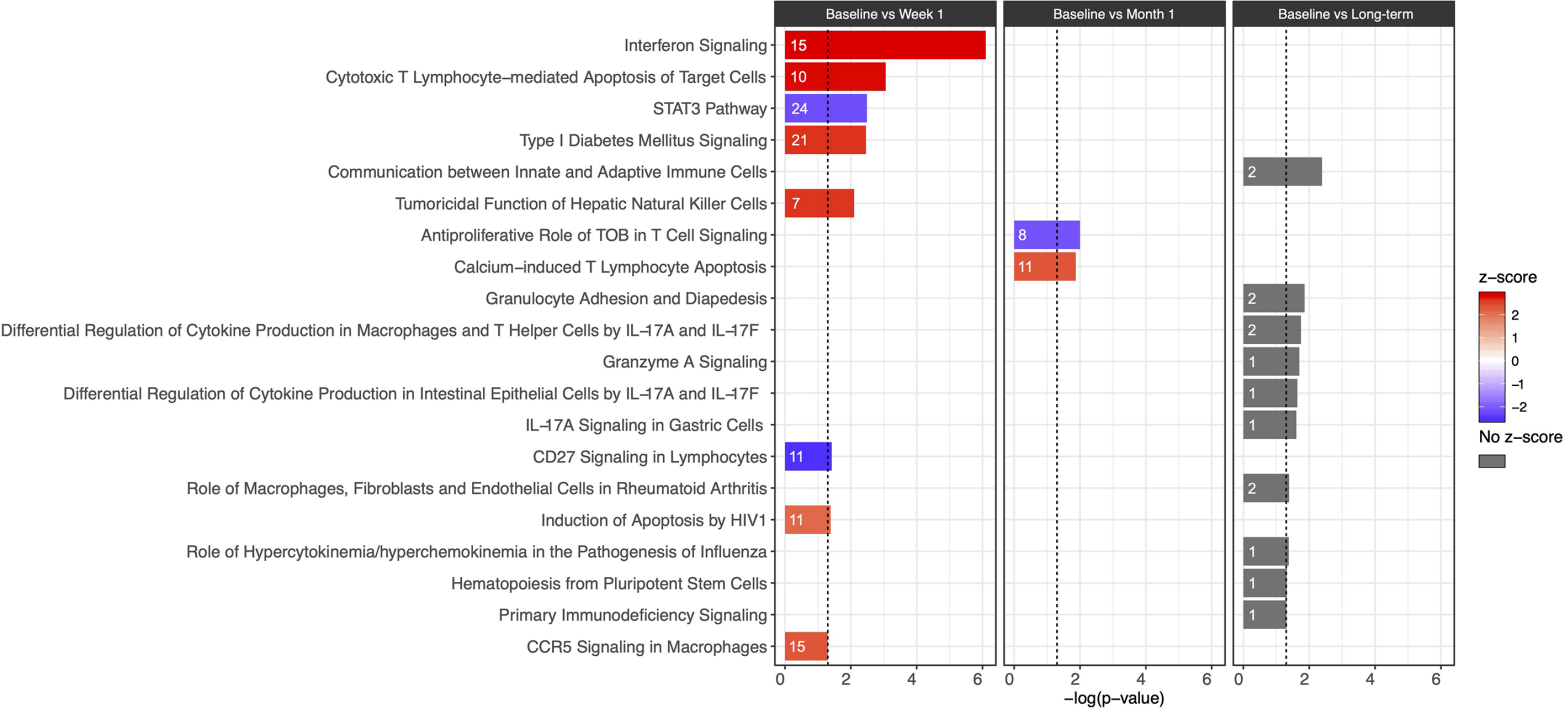
Bar plot of IPA canonical pathway analysis results comparing baseline and week 1 (first column), baseline and month 1 (second column), and baseline and long-term (third column) for immunologic pathways. The significance of an enriched pathway is represented by the length of each bar, where length corresponds to the -log(p-value). The number of genes enriching for a pathway is indicated in the bar. The vertical dotted black line corresponds to a p-value of 0.05. Positive IPA Z-scores are indicated in red, and negative Z-scores are indicated in blue. Gray coloring is used for pathways in which direction of change cannot be confidently predicted.

Evaluation of canonical pathways by IPA revealed that the DEGs from baseline *versus* week 1 significantly enriched (p ≤ 0.05, |z-score| ≥ 2) for 18 pathways, notably including pathways involved in the innate immune response and CD8+ T cell function. These included increases in *Interferon Signaling* (-log(p) = 6.1, z-score = 2.84), *Cytotoxic T Lymphocyte-mediated Apoptosis of Target Cells* (-log(p) = 3.06, z-score = 2.83), *Tumoricidal Function of Hepatic Natural Killer Cells* (-log(p) = 2.1, z-score = 2.65), and *CCR5 Signaling in Macrophages* (-log(p) = 1.3, z-score = 2.33) (**Figure 2**; see **Supplementary Datas 2** and **3** for a full listing of pathways and genes for each pathway). In contrast, decrease in relative gene expression was noted in the *STAT3 Pathway* (-log(p) = 2.49, z-score = -2.18) and *CD27 Signaling in Lymphocytes* (-log(p) = 1.42, z-score = −2.53) (**Figure 2** **).** Pathways important for apoptosis continued to be upregulated 1 month after CMV DNAemia, with enrichment in DEGs from *Calcium-Induced T Lymphocyte Apoptosis pathway* (-log(p) = 1.87, z-score = 2.33) (**Figure 2**; see **Supplementary Datas 2** and **3** for a full listing of pathways and genes for each pathway). In contrast, an important pathway related to T cell function was downregulated, namely, *Antiproliferative Role of TOB in T Cell Signaling* (-log(p) = 2, z-score = −2.12).

Although it was not possible to clearly differentiate the direction of change and assign z-score for the long-term timepoint, several important pathways were identified as undergoing significant change after resolution of DNAemia (**Figure 2**; see **Supplementary Datas 2** and **3** for a full listing of pathways and genes for each pathway). This timepoint was notable for changes in pathways important for innate immune and cytotoxic T cell function, including *Communication between Innate and Adaptive Immune cells* (-log(p) = 2.39), *Granulocyte Adhesion and Diapedesis* (-log(p) = 1.86), and *Granzyme A Signaling* (-log(p) = 1.7) (**Figure 2**). Long-term impact on T cell function is reflected by the several pathways related to IL-17 signaling, as well as relative changes in the *Hematopoiesis from Pluripotent Stem Cells* (-log(p) = 1.32) and *Primary Immunodeficiency Signaling* (-log(p) = 1.31) pathways. These results may suggest that in the long-term after resolution of CMV DNAemia, both the innate and adaptive immune responses are impacted.

### 3.4 Evaluation of Differentially Expressed Genes at 1 Week Post-DNAemia Across All Timepoints

**Figure 3:**
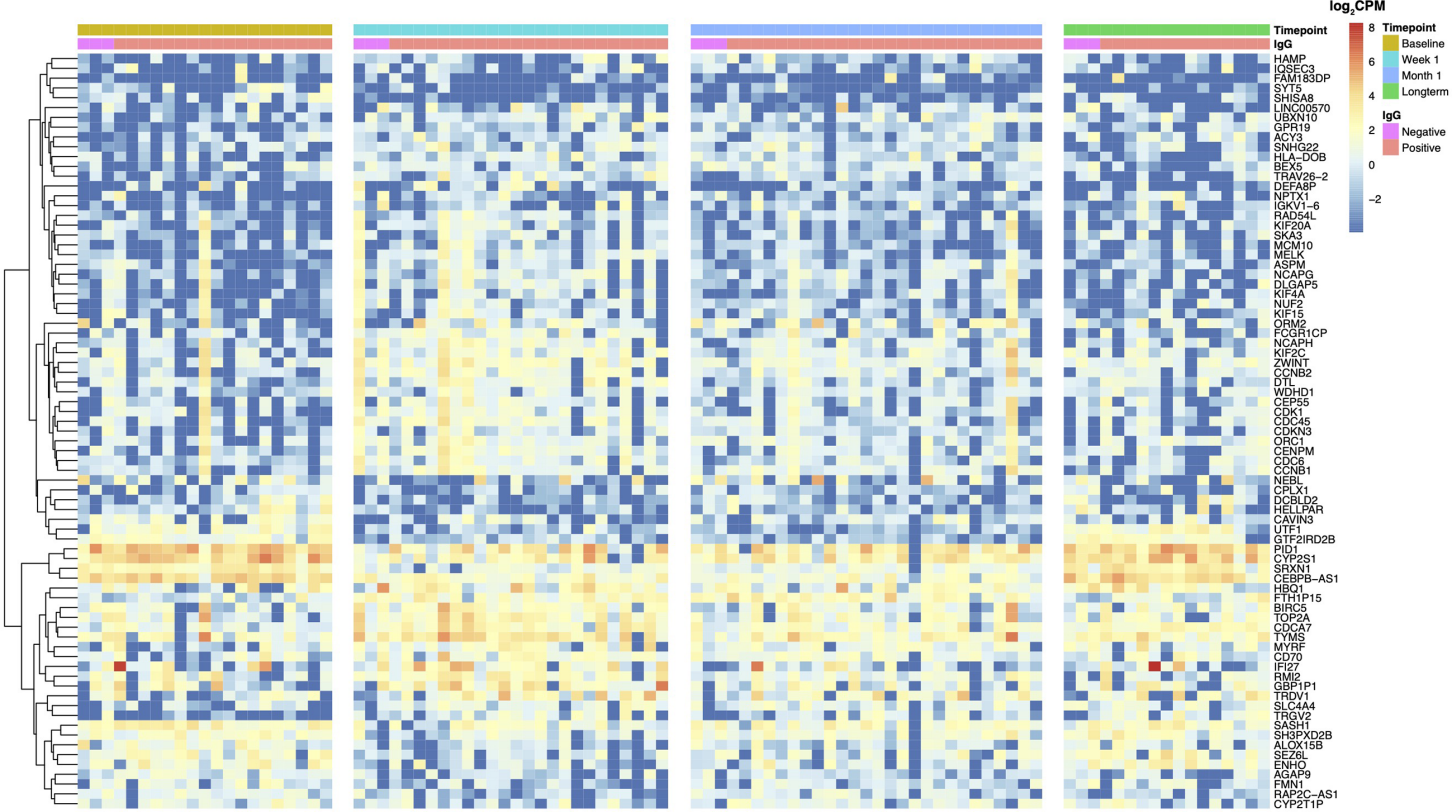
Heatmap of clustered DEGs across all timepoints for patients with CMV viremia. DEGs with |logFC| ≥ 2 at baseline *versus* week 1 post-viremia are shown. Red indicates increased expression, while blue indicates decreased expression compared with baseline. CMV IgG status of transplant recipient is indicated, with high-risk patients (D+/R−) shown in pink and intermediate-risk (R+) in orange. Timepoints are indicated as baseline (brown), week 1 (turquoise), month 1 (blue), or long-term (green) in bars at the top of the figure. Each column represents an independent patient sample.

To further visualize longitudinal changes in gene expression post-CMV DNAemia, we generated a heatmap of the logCPM expression of the DEGs between baseline and week 1 in PCR+ patients with |logFC| ≥ 2 mapped across the four timepoints (baseline, week 1, month 1, and long-term) (**Figure 3**). We observed multiple visible trends of expression, with many genes becoming either highly up- or downregulated at week 1. There is a striking difference between baseline and week 1, with multiple genes upregulated at week 1 including *FCGR1CP*, *NCPAH*, and *KIF2C*. Interestingly, at month 1 post-viremia, this upregulation is beginning to decrease, so that there is a return to near-baseline levels at the long-term sample although notably, as with the pathway analysis, expression levels for individual transcripts do not return exactly to previous levels, leading to the establishment of a new baseline for transcription after DNAemia resolution (**Figure 3**).

### 3.5 Time-Course Gene Set Analysis

**Figure 4:**
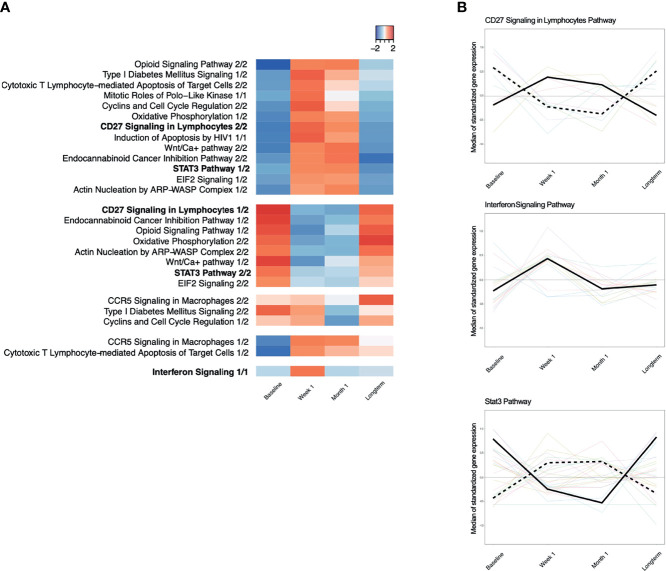
**(A)** Time-course dynamics of IPA canonical pathways significantly enriched for with genes that were differentially expressed between baseline and week 1 post-viremia. Red indicates that the median of standardized gene expression of genes in a pathway are >0, while blue indicates that the median of standardized gene expression of genes in a pathway are <0. Each column represents a different time point, namely, baseline, week 1, month 1, and long-term. Most pathways have two significant time-trend as identified by time-course gene set analysis because some genes in a given pathway have a positive median standardized gene expression, while the other genes in the same pathway have a negative median standardized gene expression. **(B)** Examples of significantly enriched IPA pathways with two time trends, CD27 Signaling in Lymphocytes Pathway and Stat3 Pathway. Interferon Signaling Pathway has only one time trend.

To determine which genes demonstrated persistent longitudinal changes, we evaluated time-trends across all four time points using TcGSA. We found that 15 of the 18 canonical pathways from baseline *versus* week 1 had a statistically significant time-trend across all four timepoints (p ≤ 0.05) (**Figure 4A** **;** **Table 3**). We observed two major patterns of gene expression: (1) downregulation of a pathway at the week 1 timepoint, followed by a gradual upregulation to the long-term timepoint or (2) upregulation at week 1 followed by a gradual downregulation to the long-term timepoint. We also observed that most of the pathways contain genes that exhibit both patterns, which leads to there being more than one significant time-trend detected by TcGSA. For example, *CD27 Signaling in Lymphocytes* and *Oxidative Phosphorylation* show two time-trends, while *Interferon Signaling* only has one time-trend (**Figure 4B**). We did not observe any significant time-trends for any of the canonical pathways identified as changing significantly from week 1 *versus* month 1 or month 1 *versus* long-term timepoints.

**Table 3:** Top 3 up/downregulated genes from baseline *vs* week 1 enriching for IPA pathways.

IPA pathway	Top 3 downregulated genes from baseline *vs* week 1	Top 3 upregulated genes from baseline *vs* week 1
**Oxidative phosphorylation**	MT-ND5, MT-ND4L, MT-ND1	NDUFA4, COX7B, NDUFB6
**EIF2 signaling**	RPS2, PIK3R6, INSR	RPL39, RPS12, RPSA
**Cyclins and cell cycle regulation**	HDAC10, HDAC9, PPM1L	CCNB2, CCNB1, CDK1
**Interferon signaling**	IFNGR2, PIAS2, TYK2	IFNG, IFIT3, ISG15
**Cytotoxic T lymphocyte-mediated apoptosis of target cells**	-	CD3D, GZMB, CD247
**Type I diabetes mellitus signaling**	TNF, IFNGR2, HLA-DMB	HLA-DOB, IFNG, CD3D
**CCR5 signaling in macrophages**	CACNA2D3, CD4, GNAI2	CD3D, MAPK11, CCR5
**Actin nucleation by ARP-WASP complex**	BAIAP2, WAS, PPP1R12C	RHOH, ARPC5L, ARPC4
**Mitotic roles of polo-like kinase**	SMC1A, PPM1L, TGFB1	CCNB2, CCNB1, CDK1
**Induction of apoptosis by HIV1**	TNF	SLC25A4, CASP3, BAK1
**Endocannabinoid cancer inhibition pathway**	CREBBP, TCF7L2, TCF4	CREB3L4, CASP3, CASP7
**STAT3 pathway**	IL1A, MAP3K21, MAP3K10	IL12RB2, MAPK11, IL18RAP
**Opioid signaling pathway**	CACNA2D3, PLCB1, RPS6KA4	RGS1, CREB3L4, PRKCH
**CD27 signaling in lymphocytes**	MAP3K10, MAP3K6, MAP3K11	CD70, CASP3, BID
**Wnt/Ca+ pathway**	FZD2, PLCB1, CREBBP	CREB3L4

### 3.6 Transcriptional Differences Between At-Risk Patients With and Without CMV DNAemia

To compare the transcriptional profile of patients who developed CMV DNAemia with those that did not develop DNAemia post-transplant, we also performed RNA-seq on 31 matched kidney transplant recipients without CMV DNAemia. We carried out differential expression tests between the following sets: (1) DNAemia *versus* non-DNAemia at baseline and (2) DNAemia *versus* non-DNAemia at the long-term timepoint.

We identified a total of 16 DEGs at FDR ≤ 0.1 with six of those DEGs having an |logFC| ≥ 1 between patients who did or did not develop DNAemia at the baseline timepoint, prior to clinical detection of CMV by PCR testing (**Table 4** **;** see **Supplementary Data 1** for a full differential expression analysis results). One of the top upregulated genes for CMV DNAemia patients at baseline is *USP18*, a deubiquitinating protease that may play a role in interferon responsiveness. Interestingly, we could also detect *PDE5A*, associated with modulation of chronic inflammation derived myeloid-derived suppressor cells and regulatory T cells activity. We also noticed significant upregulation of *PPP1R10* (protein phosphatase 1 regulatory subunit 10) and the transcription factor *NFAT5*, which regulated type 1 interferon responses, within the CMV DNAemia group at baseline. However, *SIGIRR* (Single‐immunoglobulin interleukin‐1 receptor–related), an inhibitor of TLR and IL‐1R signaling, showed significant downregulation.

**Table 4:** Differential expression results for DNAemia *versus* no DNAemia at baseline and at the long-term.

Comparison (A *vs* B)	Total DEGs*	Upregulated in B	Downregulated in B	|log_2_FC| ≥ 1
**DNAemia *vs* no DNAemia at baseline**	16	7	9	6
**DNAemia *vs* no DNAemia at long-term**	1,420	699	721	242

*FDR ≤ 0.1.

**Figure 5:**
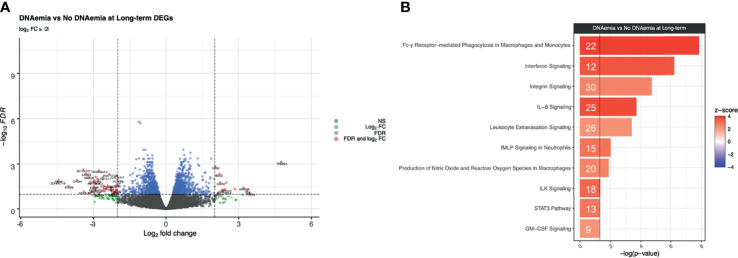
Differential expression analysis at the long-term timepoint between DNAemia and no DNAemia. **(A)** Volcano plot of differentially expressed genes. Gray points represent genes that are not differentially expressed, green points represent genes that have a log_2_FC ≥ 2 but not differentially expressed, blue points represent genes that are differentially expressed (FDR ≤ 0.1) but have a log_2_FC < 2, and red points represent genes that are both differentially expressed (FDR ≤ 0.1) and have a log_2_FC ≥ 2. **(B)** Bar plot of IPA canonical pathways enriched for patients with history of CMV DNAemia compared with those without history of DNAemia at the long-term time point. The significance of an enriched pathway is represented by the length of each bar, where length corresponds to the -log(p-value). The number of genes enriching for a pathway is indicated in the bar. The vertical dotted black line corresponds to a p-value of 0.05. Positive IPA Z-scores are indicated in red.

In contrast to the results from the baseline timepoint, there were a greater number of statistically significant differences observed at the long-term timepoint. There were 1,420 DEGs between patients with and without history of CMV DNAemia at the long-term timepoint at FDR ≤ 0.1 (**Table 4** and **Figure 5A** **)**. We observed 21 canonical pathways that were significantly enriched for (p ≤ 0.05, |z-score| ≥ 2) including multiple pathways important in innate cell functioning. These included *Fcγ Receptor-mediated Phagocytosis in Macrophages and Monocytes* (-log(p) = 7.84, z-score = 4.02), *Interferon Signaling* (-log(p) = 6.22, z-score = 3.67), and *IL-8 Signaling* (-log(p) = 3.71, z-score = 3.67) (**Figure 5B** **;** see **Supplementary Data 2** and **C** for a full listing of pathways and genes for each pathway). Interestingly, all immunologic IPA pathways demonstrating significant changes were in the direction of upregulation in patients with history of DNAemia compared with those without, which persisted despite the fact that for all patients in this cohort, DNAemia had resolved at the time of the long-term timepoint.

## 4 Discussion

This study surveyed the transcriptomic landscape of longitudinally collected peripheral blood leukocytes from kidney transplant recipients with and without CMV DNAemia. Kidney transplantation represents a unique opportunity to study the time course of CMV reactivation or primary infection given the administration of immunosuppression and close clinical monitoring for CMV by PCR in peripheral blood.

A major finding of this study is the early upregulation of gene expression followed by long-term changes in gene transcription from peripheral blood cells in patients experiencing DNAemia post-transplantation, which are not observed in patients that do not experience CMV DNAemia. These changes occurred early after DNAemia, with significant impact on transcription as early as 1 week after DNAemia start, but also persisted following resolution of DNAemia. There were detectable differences in gene expression in patients with DNAemia at the long-term compared with the baseline time point (**Figure 1**). In addition, we detected baseline differences in gene expression in patients with history of DNAemia compared with those without history of DNAemia (**Figure 5**). These changes include genes important in the innate immune response as well as T cell activation and cytotoxicity, confirmed at the individual gene level as well as *via* canonical pathway analysis.

The changes in gene expression were most striking at the 1-week post-viremia timepoint as compared to the baseline sample. These included T cell specific transcripts *TRGV2* and *TCRDV2* important in T cell receptor expression including in gamma delta T cells, and *LAG3*, *CTLA4*, and *KLRG1*, associated with T cell exhaustion. At the 1-month time point, other genes important in adaptive and innate immune function were noted including *CCL4* and *IFNG*, as well as *FCLR6* and *GPR56*, which are associated with immune aging.

While it is not surprising that the biggest impact was seen at this early timepoint, several genes were persistently altered following CMV DNAemia, leading to development of a new baseline for gene expression after resolution of CMV DNAemia. Genes in pathways such as *CCR5 Signaling in Macrophages* and *Cytotoxic T Lymphocyte-mediated Apoptosis of Target Cells* remain upregulated even after resolution of DNAemia, as shown in **Figure 2**. Of note, *Interferon Signaling* and the *STAT3 Pathway* are pathways that are downregulated in patients experiencing CMV DNAemia, yet still upregulated 9–18 months post-DNAemia relative to the patients who never experienced CMV DNAemia. Many of the individual genes seen early post-CMV DNAemia, such as *TRGV2*, *FCLR6*, and *CCL5*, remain elevated at the long-term timepoint. In addition, *PPP1R10* is known to play a role in cell cycle progression, DNA repair, and apoptosis by negatively regulating the activity of protein phosphatase 1, a proapoptotic activator important in DNA repair. This suggests that the experience of CMV DNAemia, even after resolution, leaves a lasting impact on the immune system, which promotes inflammation and immune dysregulation. Interestingly, while six patients did experience two or more episodes of CMV DNAemia, these subsequent events were generally shorter, had lower viral loads than the index event, and did not meaningfully affect gene expression profiles when DNAemia recurrence was adjusted for in differential expression analysis (data not shown).

**Figure 6:**
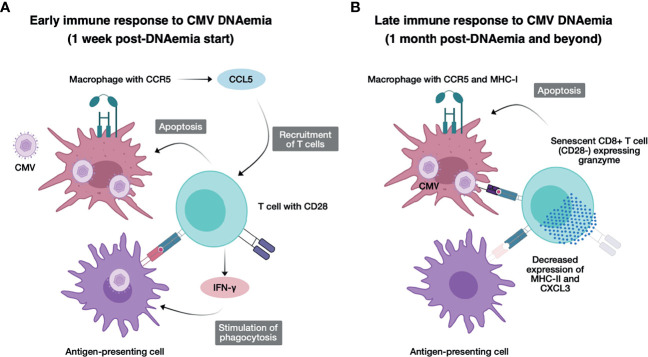
Model for immunological changes early and late after CMV DNAemia. **(A)** Concept figure illustrating the early immune response to CMV DNAemia. Cells infected with CMV including macrophages expressing CCR5 secrete CCL5, recruiting additional T cells, and undergo apoptosis from activated CD28+ T cells, which secrete IFN-g and stimulate phagocytosis in antigen-presenting cells. **(B)** Concept figure illustrating the late immune response to CMV DNAemia. Persistently infected cells including macrophages continue to present CMV antigen to CD8+ T cells, which have now become senescent, expressing LAG3 but not CD28−, and activating, expressing granzyme and triggering apoptosis in infected cells. Antigen-presenting cells have function impaired by decreased expression of MHC class II and CXCL3.

A positive association between CMV infection or reactivation has previously been shown by epidemiologic studies demonstrating increased rates of rejection and graft loss in high-risk as compared with intermediate- or low-risk kidney transplant recipients months or years after initial infection, despite receipt of antiviral prophylaxis (19). Moreover, CMV infection/reactivation can cause dysregulation in immune system (20, 21). This imbalance in the immune system may increase the risk of transplant rejection (2). Notably, many of the genes identified in our analysis as upregulated at 1 month after CMV DNAemia begins have also been identified in association with antibody-mediated or T cell-mediated rejection, including *CCL4*, *IFNG*, and *TRDV3*, as well as *FGFBP2*, which is elevated at the long-term timepoint compared with baseline timepoint (**Table 2B**). Upregulation of these genes may increase inflammation and rejection risk in the transplant allograft, namely, chemokines and chemokine receptors, and cytokines such as IL-6 (22). Other gene pathways including those impacting CCR5 have been previously identified as important in pathogenesis of rejection and chemotaxis of immune cells to the allograft (23). This observation, therefore, suggests a model by which the CMV response leads to the generation of heterologous antigen-specific T cells with cross-reactive alloimmune specificity to donor HLA molecules that can mediate allograft injury (24–26). Thus, a polyclonal antigen-specific immune response to CMV induces pro-inflammatory transcriptional upregulation in peripheral blood cells that peak early after CMV infection, but even after resolution of DNAemia, there are long-term changes in gene expression in peripheral blood cells that cause a longstanding impact in the kidney allograft (**Figure 6A**). Given that this association between CMV infection and rejection is seen in a variety of transplant recipient types (27), it is likely that this model may hold for other solid organ transplant recipients, and may explain the hypothesized connection between CMV and heart disease and other comorbidities (28–31).

The other notable finding is the lasting changes in adaptive immune response, impacting T cell senescence and exhaustion likely promoted by the fact that after resolution of clinically detectable viremia, the subclinical or latent virus triggers persistent CMV antigen exposure (**Figure 6B**). We hypothesize that in contrast to the pro-inflammatory effect of early DNAemia, this long-term effect is the likely mechanism behind vulnerability to other secondary infections including opportunistic bacterial and fungal infections that have been observed in transplant recipients (32–34). In our analysis, we noted upregulation of transcription factors associated with exhaustion including *HOPX*, which has been shown to be highly upregulated in terminally differentiated effector/memory Th1 cells and positively regulate effector differentiation, function, persistence, and survival of T cells (35). We additionally noted upregulation of *Batf2*, a transcription factor that belongs to the basic leucine zipper transcription factor family, which includes *BATF* and *BATF3*. *BATF2* is expressed in immune cells such as T cells, B cells, macrophages, and dendritic cells and IFN-γ–inducible *BATF2* in innate immune cells controls Th17-mediated immunopathology by suppressing IL-23 production, as well as *FCRL6*, which is upregulated in CD56dim NK cells, Vdelta1+ and Vdelta2+ gamma-delta T cells, effector and effector memory CD8+ T cells, and rare cytotoxic CD4+ T cells in diseases characterized by chronic immune stimulation (36). This pro-inflammatory process likely drives CMV and aging-associated T cell senescence, leading to premature increase in frequency of senescent and exhausted T cells in individuals with history of CMV exposure, even in healthy adults without end-organ disease or history of transplantation. Understanding the mechanisms behind the deleterious effects of CMV at different timepoints of infection can suggest possible interventions, and ideal timing of interventions, to block these negative outcomes.

Many attempts have been made to establish risk stratification for CMV DNAemia as a tool in organ transplantation, including detection of a CMV-specific T cell response (37, 38). We found that at the baseline sample prior to CMV DNAemia, there were already differences in gene expression between those who developed *versus* those who never develop CMV DNAemia (**Table 4** **;** **Supplementary Data 1**). One of these genes, *USP18*, is related to interferon responsiveness (39), raising the question of whether this indicates increased vulnerability to CMV, as opposed to the detection of low levels of CMV replication below the limit of detection of the clinical PCR test. Further exploration of this observation may reveal more about the mechanism behind vulnerability to CMV, especially reactivation of CMV in CMV seropositive patients. In addition, it suggests the possibility of using monitoring of host transcription for early diagnosis and risk stratification of CMV infection or reactivation.

One limitation of this study is that most patients, in both the CMV DNAemia and no CMV DNAemia arms, have a history of CMV infection. Therefore, it is likely that the majority of the transcriptomic profiles of CMV DNAemic patients represent CMV reactivation and not primary infection. This issue also makes it difficult to compare the impact of high risk (D+/R−) status *versus* intermediate risk (R+) status on acute and long-term changes in gene expression. Another potential limitation of this study is the fact that all patients received antiviral therapy after CMV DNAemia was detected, while the patients without CMV DNAemia received antiviral prophylaxis and monitoring only. However, this limitation is mitigated by the fact that as a single-center study, all patients received similar regimens of immunosuppression, monitoring for rejection, antiviral prophylaxis, and CMV PCR monitoring. In addition, the most striking differences in gene expression were observed early after CMV DNAemia, likely before the start of antiviral therapies, which in addition would be unlikely to directly affect gene expression. We additionally lacked data on vaccination in this cohort, which would have largely consisted on influenza vaccine during this time period; however, we would predict that the impact of CMV viremia would overshadow any vaccination impact, which would have occurred in similar frequency in the CMV infection compared with the control patient cohorts. Another limitation of this study is the lack of associations between study participants and favorable or adverse clinical outcomes because of the relatively small size of our cohort and the low incidence of either favorable or adverse clinical events. We plan to perform future studies with a case-control study design that can properly address this limitation. Yet another limitation to this analysis is that as patients are followed longitudinally over the course of an episode of CMV DNAemia, they are also progressively farther away from induction immunosuppression, which may also have an impact. One meliorating factor, however, is that the control group is also followed over the year after transplantation, allowing analysis of changes related to CMV infection as opposed to change in time from transplantation alone. Finally, as we performed bulk RNA-seq on whole PBMC samples, we are not able to characterize the longitudinal transcriptomic profiles of specific cell populations. In future studies, we plan to perform transcriptomic profiling of CMV-tetramer positive T cells or single-cell RNA-seq to overcome this limitation.

These data provide new insights into *in vivo* dynamics of the longitudinal immune response to CMV DNAemia, which have the potential to identify mechanisms of susceptibility to CMV infection and allograft rejection.

## Data Availability Statement

The datasets presented in this study can be found in online repositories. The names of the repository/repositories and accession number(s) can be found below: https://www.ncbi.nlm.nih.gov/geo/query/acc.cgi?acc=GSE168598.

## Ethics Statement

The studies involving human participants were reviewed and approved by UCLA Institutional Review Board. The patients/participants provided their written informed consent to participate in this study.

## The CMV Systems Immunobiology Group

Richard Ahn, Janice Arakawa-Hoyt, Patrick Boada, Jenny Brook, Suphamai Bunnapradist, Jim Cimino, Izabella Damm, Nakul Datta, Mario Deng, Don J. Diamond, Tin Doung, Janette Gadzhyan, David Elashoff, David Gjertson, Victoria Groysberg, Alexander Hoffmann, Kenichi Ishiyama, Maggie Kerwin, Lewis L. Lanier, Megan Llamas, Erik Lum, Dane Munar, Tariq Mukatash, Harry Pickering, Zachary Qian, Michelle Ramirez, Elaine F. Reed, Priyanka Rashmi, Rodney Rodgers, Dimitri Rychov, Minnie M. Sarwal, Joanna Schaenman, Subha Sen, Tara Sigdel, Danielle Sim, Marina Sirota, Swastika Sur, Otto Yang.

## Author Contributions

ER, AH, MR, and DG designed the study. RA and JS drafted the manuscript. HP, SS, MR, DG, MD, DD, MS, and ER provided critical feedback for the manuscript. RA and ZQ performed all data preprocessing and analyses. MR was responsible for generating RNA-seq data. VG, ML, and SB provided demographic and clinical data. All authors contributed to the article and approved the submitted version.

## Funding

This study was supported by a National Institutes of Health grant U19 AI128913 (PIs: ER and MS). The funding bodies played no role in the collection, analysis, or interpretation of data in this study.

## Conflict of Interest

The authors declare that the research was conducted in the absence of any commercial or financial relationships that could be construed as a potential conflict of interest.

## Publisher’s Note

All claims expressed in this article are solely those of the authors and do not necessarily represent those of their affiliated organizations, or those of the publisher, the editors and the reviewers. Any product that may be evaluated in this article, or claim that may be made by its manufacturer, is not guaranteed or endorsed by the publisher.
