# Supplementary material for: Acute and Chronic Changes in Gene Expression After CMV DNAemia in Kidney Transplant Recipients
Source: Front Immunol. 2021 Nov 15;12:750659. doi: 10.3389/fimmu.2021.750659 (PMC8634678; doi:10.3389/fimmu.2021.750659)
Supplement: Supplementary file 3 [file Table_3.docx]

**Supplementary Data. Full listing of genes from each significantly enriched pathway.**

**Baseline vs Week 1 in DNAemia samples**

| **Ingenuity Canonical Pathways** | **Genes** |
| --- | --- |
| Oxidative Phosphorylation | NDUFB9, MT-CYB, COX7B, MT-ND5, NDUFA12, MT-ND2, MT-ND1, SDHB, COX7A2, COX17, COX6B1, SDHD, MT-ND4L, NDUFS5, UQCRFS1, ATP5F1B, MT-ND4, ATP5F1E, MT-ATP6, MT-CO1, NDUFB3, COX7A2L, SURF1, NDUFA2, ATP5F1D, NDUFAB1, NDUFS1, COX6C, NDUFA4, NDUFB6, NDUFS2, UQCRQ, ATP5MG, ATP5PD, COX8A, NDUFB1, MT-CO2, NDUFB4, UQCRB, NDUFB2, COX7C |
| EIF2 Signaling | RPL7A, PIK3R6, RPL32, RAP1A, MT-TM, RPS13, RALB, RPS19, RPL14, RPL30, RPL39, RPS6, EIF3A, PABPC1, RPS7, INSR, RPS12, IGF1R, RPS16, RPS9, EIF3K, RPS27A, RPL15, RPL27, EIF3F, RPS27L, UBA52, RPS27, EIF4A2, RPL35A, RPS20, RPL6, RPL5, EIF2S2, RPL38, RPL37, RPL3, RPL22L1, RPL31, RPS8, RPS3A, EIF4A3, RPS21, EIF4G1, RPS2, AKT2, RPS14, RPSA, RPL24, MAP2K2, RPL36AL, RPL34, RPS23, EIF4E, RPL10, RPL10A |
| Cyclins and Cell Cycle Regulation | TGFB1, CCND2, HDAC10, CDK2, PPM1L, E2F1, HDAC3, CCNB2, PPP2R5C, CCNH, SKP1, CCNA2, RB1, HDAC9, CCNB1, CDK1 |
| Interferon Signaling | IFITM1, IFNG, IFNGR2, IFIT3, IFI6, IFITM3, PIAS1, TAP1, TYK2, BAK1, IFIT1, STAT1, ISG15, BAX, PSMB8 |
| Cytotoxic T Lymphocyte-mediated Apoptosis of Target Cells | CD3G, CD3D, PRF1, CASP3, B2M, HLA-B, CD247, BID, CASP7, GZMB |
| Type I Diabetes Mellitus Signaling | MAPK14, IFNG, IFNGR2, CD3G, PRF1, HSPD1, HLA-G, HLA-DOB, PIAS1, HLA-F, GZMB, STAT1, TNF, CD3D, HLA-DMB, CASP3, MAPK11, HLA-B, BID, CD247, RIPK1 |
| Tumoricidal Function of Hepatic Natural Killer Cells | SRGN, PRF1, CASP3, BAX, BID, CASP7, GZMB |
| CCR5 Signaling in Macrophages | MAPK14, CCL5, CALM1, CD3G, PRKCQ, GNG10, GNAI3, CCR5, GNAI2, CD3D, CACNA2D3, CD4, MAPK11, PRKCH, CD247 |
| Actin Nucleation by ARP-WASP Complex | RHOH, CDC42, ARPC3, WIPF1, ARPC5L, PPP1R12C, RAP1A, RHOA, BAIAP2, RALB, WAS, ARPC4, NCK1, ARPC1A |
| Mitotic Roles of Polo-Like Kinase | TGFB1, KIF23, ESPL1, HSP90B1, SMC1A, PPM1L, PRC1, CDC20, CCNB2, ANAPC11, PPP2R5C, CDC7, ANAPC5, RAD21, PKMYT1, CDC16, HSP90AB1, HSP90AA1, PTTG1, CCNB1, CDK1 |
| Induction of Apoptosis by HIV1 | BIRC2, SLC25A5, TNF, HTRA2, SLC25A4, CASP3, BAX, BID, RIPK1, BAK1, SLC25A3 |
| Endocannabinoid Cancer Inhibition Pathway | MAP2K5, MAPK14, AKT1S1, PIK3R6, TCF3, CCND2, CREB1, TCF4, AKT2, GNAI3, RHOA, VIM, GNAI2, GNAQ, RPTOR, CREB5, CREB3L4, MAP2K2, CASP3, CREBBP, TCF7L2, PRKACA, PRKAG1, CASP7 |
| STAT3 Pathway | IL21R, MAPK14, MAP3K20, TGFB1, MAP3K11, CSF2RB, IL12RB2, INSR, MAP3K10, RAP1A, TYK2, IGF1R, RALB, BMPR2, IL17RA, MAP3K21, IL18RAP, IL1A, MAP2K2, MAPK11, HGF, IL17RC, IL18R1, IL12RB1 |
| Opioid Signaling Pathway | MAP2K5, CDC42, CALM1, CREB1, PRKCQ, RAP1A, GNAI3, AP1B1, RALB, GNAI2, LCK, CACNA2D3, MAPK7, SIGMAR1, CREBBP, PRKCH, RPS6KA4, PLCB1, EP300, PPP3CB, PPP3CA, AKT2, BLK, CREB5, RGS1, CREB3L4, SRF, RGS14, CAMK1, AP2A2, MAP2K2, PLD2, AP2A1, PRKAG1, PRKACA |
| FAT10 Cancer Signaling Pathway | BMPR2, IFNG, TGFB1, TNF, TCF4, PCNA, AKT2, MAD2L1, ACVR2B |
| D-myo-inositol (1,4,5)-Trisphosphate Biosynthesis | PLCB1, PLCB2, PLCB3, PLD4, PIP5K1C, PI4KA |
| CD27 Signaling in Lymphocytes | MAP2K5, SIVA1, MAP3K11, MAP3K1, MAP2K2, MAP3K6, MAP3K10, CASP3, BID, CD70 |
| Wnt/Ca+ pathway | PLCB1, CREB1, PLCB2, FZD2, PPP3CA, NFATC1, DVL1, CREB3L4, CREB5, PLCB3, CREBBP, NFAT5, DVL3 |

**Baseline vs Month 1 in DNAemia samples**

| **Ingenuity Canonical Pathways** | **Genes** |
| --- | --- |
| AMPK Signaling | ADRB2,AKT1S1,AKT2,ARID1A,CREB1,CREB3,CREBBP,CRTC2,EIF4EBP1,EP300,HMGCR,INSR,MAPK1,PIK3R6,PPP2CA,PPP2R1B,PPP2R5C,PRKACA,RAB11A,SMARCA4,SMARCC2,SMARCD3,STK11,STRADA,TSC2 |
| Calcium-induced T Lymphocyte Apoptosis | CABIN1,CALM1,CD247,CD3D,CHP1,EP300,HDAC1,HLA-B,HLA-DOB,NR4A1,PRKCH |
| Antiproliferative Role of TOB in T Cell Signaling | CD247,CD3D,DPP7,MAPK1,SKP1,TGFB1,TGFBR1,TOB1 |
| EIF2 Signaling | AKT2,ATF3,ATF5,EIF3F,EIF3K,EIF4G1,INSR,MAP2K2,MAPK1,MT-TM,PIK3R6,PPP1R15A,RAP1A,RASD2,RPL10,RPL10A,RPL15,RPL22L1,RPL23,RPL27,RPL3,RPL30,RPL31,RPL34,RPL35A,RPL36AL,RPL37,RPL38,RPL5,RPL6,RPS12,RPS13,RPS14,RPS19,RPS2,RPS20,RPS23,RPS27A,RPS27L,RPS6,RPS7,RPS8,RPS9,RPSA,RRAS,SREBF1,UBA52,VEGFA |
| Oxidative Phosphorylation | ATP5F1A,ATP5F1B,ATP5F1D,ATP5F1E,ATP5MC1,ATP5MG,ATP5PD,COX6C,COX7A2,COX7A2L,COX7B,COX7C,MT-ATP6,MT-CO2,MT-ND1,MT-ND2,MT-ND4,MT-ND4L,MT-ND5,NDUFA2,NDUFA3,NDUFA4,NDUFA6,NDUFB1,NDUFB10,NDUFB2,NDUFB4,NDUFB6,NDUFB9,NDUFS1,NDUFS3,NDUFS5,SDHC,UQCRB,UQCRC2,UQCRFS1,UQCRQ,VPS9D1 |

**Baseline vs Longterm in DNAemia samples**

| **Ingenuity Canonical Pathways** | **Genes** |
| --- | --- |
| Communication between Innate and Adaptive Immune Cells | CCL5,IGHA1 |
| Sphingosine-1-phosphate Signaling | ADCY4,PDGFD |
| Renin-Angiotensin Signaling | ADCY4,CCL5 |
| Pathogenesis of Multiple Sclerosis | CCL5 |
| Granulocyte Adhesion and Diapedesis | CCL5,CLDN9 |
| Hepatic Fibrosis / Hepatic Stellate Cell Activation | PDGFD,CCL5 |
| Agranulocyte Adhesion and Diapedesis | CCL5,CLDN9 |
| Adrenomedullin signaling pathway | ADCY4,MATK |
| Differential Regulation of Cytokine Production in Macrophages and T Helper Cells by IL-17A and IL-17F | CCL5 |
| Granzyme A Signaling | GZMA |
| Actin Cytoskeleton Signaling | PDGFD,MATK |
| Differential Regulation of Cytokine Production in Intestinal Epithelial Cells by IL-17A and IL-17F | CCL5 |
| IL-17A Signaling in Gastric Cells | CCL5 |
| Role of Macrophages, Fibroblasts and Endothelial Cells in Rheumatoid Arthritis | PDGFD,CCL5 |
| Role of Hypercytokinemia/hyperchemokinemia in the Pathogenesis of Influenza | CCL5 |
| Serotonin Receptor Signaling | ADCY4 |
| Hematopoiesis from Pluripotent Stem Cells | IGHA1 |
| Primary Immunodeficiency Signaling | IGHA1 |

**DNAemia vs No DNAemia at Longterm**

| **Ingenuity Canonical Pathways** | **Genes** |
| --- | --- |
| Ephrin Receptor Signaling | SH2D3C, ACTR2, PAK1, LIMK1, WIPF1, RAC2, RGS3, EPHB1, EPHA4, GNB2, ARPC5, RALB, WAS, GNAI2, CFL1, GNG5, CREB3, ARPC4, ABI1, SDCBP, GRIN3A, MAPK3 |
| Fcγ Receptor-mediated Phagocytosis in Macrophages and Monocytes | HCK, RAB11A, ACTR2, TLN1, FCGR1A, PAK1, VASP, NCF1, FYB1, RAC2, FCGR2A, VAV2, ARPC5, LCP2, WAS, ACTG1, VAMP3, ACTB, ARPC4, PLD2, MAPK3, FCGR3A/FCGR3B |
| IL-8 Signaling | AZU1, IQGAP1, DEFA1, PIK3C2A, LIMK1, ARRB2, RAC2, NCF2, TEK, FOS, RALB, GNAI2, RHOT1, MAPK3, RHOT2, MYL12B, VASP, MPO, GNB2, RHOG, GNG5, CXCR2, CXCR1, PTGS2, PLD2, BCL2L1 |
| Regulation of Actin-based Motility by Rho | ACTR2, RHOT2, PAK1, MYL12B, LIMK1, MYL12A, WIPF1, RAC2, RHOG, ARPC5, MYL6, WAS, CFL1, PFN1, ACTB, GSN, ARPC4, RHOT1 |
| Interferon Signaling | IFIT1, IFITM1, IFNG, STAT1, IFITM2, ISG15, IFIT3, MX1, IFI35, TAP1, IFNAR1, PSMB8 |
| RhoA Signaling | ACTR2, ARHGAP5, MYL12B, LIMK1, MSN, MYL12A, RHPN1, ARPC5, MYL6, CFL1, ACTG1, PFN1, ACTB, ARPC4, CDC42EP2 |
| Signaling by Rho Family GTPases | IQGAP1, ACTR2, PIK3C2A, LIMK1, WIPF1, NCF2, FOS, ARPC5, WAS, GNAI2, CFL1, ARHGEF17, ARHGEF7, ACTG1, ACTB, ARPC4, RHOT1, MAPK3, RHOT2, PAK1, MYL12B, MYL12A, MSN, GNB2, RHOG, MYL6, GNG5, CDC42EP2 |
| ILK Signaling | RHOT2, PIK3C2A, TNFRSF1A, ACTN1, GSK3A, ILK, FOS, RHOG, MYL6, CFL1, ACTG1, ACTB, CREB3, PTGS2, RHOT1, ACTN4, TMSB10/TMSB4X, MAPK3 |
| fMLP Signaling in Neutrophils | ACTR2, PIK3C2A, NCF1, FPR2, PPP3CB, FPR1, GNB2, NCF2, ARPC5, RALB, WAS, GNAI2, GNG5, ARPC4, MAPK3 |
| Actin Cytoskeleton Signaling | IQGAP1, ACTR2, PIK3C2A, LIMK1, RAC2, ARPC5, RALB, WAS, CFL1, PFN1, ARHGEF7, ACTG1, ACTB, ARPC4, ACTN4, MAPK3, TLN1, CD14, PAK1, MYL12B, MYL12A, MSN, ACTN1, VAV2, MYL6, GSN, CSK, TMSB10/TMSB4X |
| Cdc42 Signaling | IQGAP1, HLA-E, ACTR2, PAK1, MYL12B, LIMK1, MYL12A, WIPF1, B2M, VAV2, FOS, ARPC5, MYL6, WAS, CFL1, ARPC4, CDC42EP2 |
| STAT3 Pathway | PIAS3, SOCS4, CSF2RB, IL11RA, IFNAR1, IL2RG, IL6ST, RALB, IL10RB, CXCR2, CXCR1, MAPK3, IL12RB1 |
| Remodeling of Epithelial Adherens Junctions | IQGAP1, ACTR2, ACTN1, ZYX, ARPC5, RAB5C, DNM1L, TUBA1A, RAB7A, RAB5B, ACTG1, ACTB, ARPC4, TUBA1B, TUBA4A, ACTN4 |
| Production of Nitric Oxide and Reactive Oxygen Species in Macrophages | NCF4, S100A8, RHOT2, IFNG, PIK3C2A, TNFRSF1A, MPO, PPP1CA, NCF1, MAP3K13, NCF2, FOS, RHOG, TLR4, STAT1, CAT, SERPINA1, RHOT1, APOL1, MAPK3 |
| Integrin Signaling | ACTR2, PIK3C2A, NEDD9, WIPF1, ILK, RAC2, ZYX, ARPC5, RALB, WAS, PFN1, ARHGEF7, ACTG1, ACTB, ARPC4, RHOT1, ACTN4, MAPK3, RHOT2, TLN1, ARHGAP5, PAK1, MYL12B, VASP, MYL12A, ACTN1, RHOG, ARF1, GSN, ARF4 |
| iNOS Signaling | TLR4, FOS, LY96, IFNG, STAT1, CD14 |
| Actin Nucleation by ARP-WASP Complex | RHOG, ARPC5, RALB, WAS, ACTR2, RHOT2, ARPC4, VASP, RHOT1, WIPF1 |
| Leukocyte Extravasation Signaling | F11R, PIK3C2A, SIPA1, NCF1, WIPF1, RAC2, NCF2, WAS, GNAI2, ACTG1, ACTB, ICAM3, ACTN4, NCF4, ARHGAP5, VASP, MSN, ACTN1, TIMP2, VAV2, MYL6, SELPLG, MMP8, PECAM1, MMP23B, MMP14 |
| GM-CSF Signaling | RALB, HCK, PIK3C2A, BCL2A1, STAT1, CSF2RB, PPP3CB, BCL2L1, MAPK3 |
| Pentose Phosphate Pathway | TKT, G6PD, PGD, TALDO1 |
| RhoGDI Signaling | ACTR2, RHOT2, ARHGAP5, PAK1, MYL12B, LIMK1, MYL12A, MSN, ARHGDIB, GNB2, RHOG, ARPC5, MYL6, GNAI2, CFL1, GNG5, ARHGEF17, ARHGEF7, ACTG1, ACTB, ARPC4, RHOT1 |
